# Lifespan trajectories of motor control and neural oscillations: A systematic review of magnetoencephalography insights

**DOI:** 10.1016/j.dcn.2025.101529

**Published:** 2025-02-09

**Authors:** Xinbi Zhang, Mingming Huang, Xiaoxia Yuan, Xiaoke Zhong, Shengyu Dai, Yingying Wang, Qiang Zhang, Kanya Wongwitwichote, Changhao Jiang

**Affiliations:** a The Center of Neuroscience and Sports, Capital University of Physical Education and Sports, Beijing 100191, China; b School of Kinesiology and Health, Capital University of Physical Education and Sports, Beijing 100191, China; c The School of Sport, Exercise and Rehabilitation Sciences, University of Birmingham, Birmingham, UK; d School of Physical Education and Sport Science, Fujian Normal University, No. 18, Wulongjiang Middle Avenue, Shangjie Town, Minhou County, Fuzhou 350108, China

**Keywords:** Magnetoencephalography, Motor control development, Neural oscillations, Movement-related oscillations, Lifespan trajectory

## Abstract

Motor control (MC) evolves across the human lifespan, improving during childhood and adolescence, stabilizing in early adulthood, and declining in older age. These developmental and degenerative patterns are linked to neural oscillatory activity, which can be assessed via magnetoencephalography (MEG) to gain insights into motor planning, execution, termination, and command initiation. This review systematically examined age-related changes in MC and neural oscillations, centering on movement-related beta desynchronization (MRBD), post-movement beta rebound (PMBR), and movement-related gamma synchrony (MRGS). Following PRISMA guidelines, 17 cross-sectional studies were identified. The findings revealed significant enhancements in motor efficiency from childhood to adolescence, characterized by faster movement speed, shorter movement duration, weaker MRBD, and increased PMBR and MRGS. From adolescence to early adulthood, further improvements in motor performance were noted, accompanied by strengthened MRBD, PMBR, and a slight decline in MRGS. In older adults, motor performance deteriorates, presenting as slower movement and prolonged duration, alongside heightened resting beta power, elevated MRBD, and reduced PMBR. Alterations in MRGS remain insufficiently explored. Overall, MEG proves valuable for capturing neural dynamics underlying the development and decline of motor control across the lifespan. These findings underscore potential avenues for motor rehabilitation and cognitive interventions, particularly in aging populations.

## 1 Introduction

Motor control (MC) refers to the ability to regulate and coordinate movements, encompassing activities ranging from simple tasks like reaching for an object to complex behaviors such as playing an instrument or engaging in sports. It is a fundamental aspect of human behavior, translating cognitive and psychological processes into physical actions (Rosenbaum et al., 2012). Throughout the human lifespan, MC follows a nonlinear developmental trajectory: it improves during childhood and adolescence, peaks in early adulthood, and gradually declines with age (Maes et al., 2017, Gaetz et al., 2010). Understanding the underlying neurophysiological mechanisms driving these changes is crucial for elucidating both the development of motor skills and age-related motor decline.

Various neuroimaging methods, including electroencephalography (EEG) (Rossini et al., 2007) and functional magnetic resonance imaging (fMRI) (Schulte et al., 2020), have been employed to explore the neural basis of MC development. However, these techniques face limitations due to trade-offs between temporal and spatial resolution, making it challenging to fully capture the real-time neural dynamics involved in motor control.

Magnetoencephalography (MEG) offers a unique solution to these limitations by providing both high temporal (∼1 ms) and spatial (∼2–5 mm) resolution. This makes MEG particularly well-suited for studying the rapid and complex neural processes underlying motor control (Brookes et al., 2022). MEG detects magnetic fields generated by synchronized neural activity, allowing precise tracking of brain function during movement (Cohen, 1968, Murakami and Okada, 2006). Unlike EEG, MEG is less affected by distortions caused by the skull, providing more accurate localization of neural oscillations involved in motor processes (Walshe et al., 2023). Consequently, MEG is a powerful tool for studying the neural oscillations that underpin motor control, particularly in the beta and gamma frequency bands, and can accurately record rich and dynamic brain activity during movement (Hedrich et al., 2017, Wens, 2023). MEG offers novel insights into critical aspects of brain activity underlying MC and its development, including the precise timing of localized activity and neural oscillations with high spatial resolution.

Three key oscillatory patterns have been shown to play central roles in motor control: movement-related beta desynchronization (MRBD), reflecting a reduction in beta power during motor planning and execution (Heinrichs-Graham et al., 2018a); post-movement beta rebound (PMBR), signaling motor inhibition and sensory feedback processes following task completion (Heinrichs-Graham and Wilson, 2016, Tzagarakis et al., 2010); and movement-related gamma synchrony (MRGS), associated with the initiation of motor commands (Cheyne and Ferrari, 2013, Heinrichs-Graham et al., 2017). These neural oscillations exhibit systematic changes across the lifespan and serve as critical markers of the neurophysiological processes underlying both motor development and decline.

Previous research has demonstrated age-related changes in these oscillatory patterns, with significant developmental shifts occurring during childhood and adolescence, early adulthood, and older age (Heinrichs-Graham et al., 2018a, Rossiter et al., 2014). This systematic review aims to investigate the changes in MC-related neural oscillations across these critical developmental stages, providing a comprehensive synthesis of MEG findings. By focusing on MRBD, PMBR, and MRGS, this review seeks to enhance our understanding of how neural oscillations evolve with motor control and aging, and to uncover the neurophysiological mechanisms driving these changes.

## 2 Methods

### 2.1 Study design

This systematic review was conducted in accordance with the guidelines outlined in the Preferred Reporting Items for Systematic Reviews and Meta-Analyses (PRISMA) statement. The review protocol was registered on the PROSPERO International Prospective Register of Systematic Reviews in April 2024 (http://www.crd.york.ac.uk/prospero/, accessed on 05 July 2024), with the registration number: CRD42024560642.

### 2.2 Eligibility criteria

Studies were included in the review if they met the following criteria: (1) involved healthy participants across distinct age groups based on the World Health Organization’s (WHO) age classification (World Health Organization, 2015)—children (4–9 years), adolescents (10–19 years), young adults (20–39 years), middle-aged adults (40–59 years), and older adults (60 years and above); (2) employed MEG to assess motor control and associated neural oscillations, specifically MRBD, PMBR, MRGS; (3) provided comparative MEG data on motor control across these age groups; (4) examined changes in neural oscillations, motor performance metrics, and related neurophysiological mechanisms; and (5) were cross-sectional studies published in peer-reviewed journals. Studies were excluded if they: (1) were longitudinal or cohort studies, reviews, meta-analyses, theoretical papers, case studies, or experimental studies involving interventions; or (2) were not published in English or did not use MEG to assess motor control.

### 2.3 Search strategy

A comprehensive literature search was performed using the following electronic databases: MEDLINE (via PubMed), Web of Science, Scopus, PsycINFO, EBSCO, and Embase. The search covered all available literature up to October 2024. The strategy incorporated a combination of keywords related to "magnetoencephalography," "motor control," "development," "aging," and key neural oscillations such as "beta desynchronization," "gamma synchrony," and "post-movement beta rebound." The specific search terms are presented in Table 1. Additionally, the reference lists of retrieved studies were screened for any further relevant literature.

**Table 1:** Search terms used to identify relevant articles in database.

1	motor control	16	PMBR
2	movement	17	MRGS
3	motor skills	18	cross-sectional
4	motor coordination	19	comparison
5	childhood	20	developmental study
6	adolescence	21	reaction time
7	adulthood	22	accuracy
8	aging	23	movement duration
9	lifespan	24	motor performance
10	magnetoencephalography		
11	MEG		
12	beta desynchronization		
13	post-movement beta rebound		
14	gamma synchrony		
15	MRBD		
25	MOTOR CONTROL SEARCH TERMS: 1 OR 2 OR 3 OR 4		
26	DEVELOPMENTAL SEARCH TERMS: 8 OR 6 OR 7 OR 8 OR 9		
27	MEG SEARCH TERMS: 10 OR 11		
28	NEURAL OSCILLATION SEARCH TERMS: 12 OR 13 OR 14 OR 15 OR 16 OR 17		
29	STUDY DESIGN SEARCH TERMS: 18 OR 19 OR 20		
30	OUTCOME SEARCH TERMS: 21 OR 22 OR 23 OR 24		
31	FINAL SEARCH TERMS: 25 AND 26 AND 27 AND 28 AND 29 AND 30		

### 2.4 Study selection and data extraction

Two independent reviewers conducted an initial screening of titles and abstracts from the identified studies based on the eligibility criteria. Full-text versions of studies deemed potentially relevant were then retrieved for further evaluation. Any disagreements between the reviewers were resolved through discussion, or, if necessary, by involving a third reviewer. Data extraction was performed using a standardized form that included key details, such as study characteristics (authors, year, country), participant demographics (sample size, age range, gender, dominant side), MEG measurement specifics (equipment, number of channels, sampling rate, data analysis techniques), journal, study design, outcome measures (both behavioral and neuronal), and the primary findings related to both behavioral and neuronal aspects.

### 2.5 Quality and risk-of-bias assessment

The included studies were assessed for risk of bias using the Appraisal Tool for Cross-Sectional Studies (AXIS) (Downes et al., 2016) (see Table 2). To quantify the risk of bias, a scoring system was adapted following the approach by Kühn et al. (2021). Studies were categorized as having a very low risk of bias if they answered 19 out of 20 questions correctly, a low risk if they scored between 17 and 18, and a moderate risk if their score was between 16 and 15, and as high risk of bias if studies scored 14 or fewer points. The AXIS tool evaluates various aspects of study quality, including design, sample representativeness, data collection, and statistical analysis.

**Table 2:** AXIS tool evaluation items.

Questions	Evaluation items
*Introduction*	
1	Were the aims/objectives of the study clear?
*Methods*	
2	Was the study design appropriate for the stated aim(s)?
3	Was the sample size justified?
4	Was the target/reference population clearly defined? (Is it clear who the research was about?)
5	Was the sample frame taken from an appropriate population base so that it closely represented the target/reference population under investigation?
6	Was the selection process likely to select subjects/participants that were representative of the target/reference population under investigation?
7	Were measures undertaken to address and categorise non-responders?
8	Were the risk factor and outcome variables measured appropriate to the aims of the study?
9	Were the risk factor and outcome variables measured correctly using instruments/measurements that had been trialled, piloted or published previously ?
10	Is it clear what was used to determined statistical significance and/or precision estimates? (eg, p values, Cls)
11	Were the methods (including statistical methods) sufficiently described to enable them to be repeated?
*Results*	
12	Were the basic data adequately described?
13	Does the response rate raise concerns about non-response bias?
14	If appropriate, was information about non-responders bias?
15	Were the results internally consistent ?
16	Were the results for the analyses described in the methods, presented?
*Discussion*	
17	Were the authors’ discussions and conclusions justified by the results?
18	Were the limitations of the study discussed ?
*Other*	
19	Were there any funding sources or conflicts of interest that may affect the authors’ interpretation of the results?
20	Was ethical approval or consent of participants attained?

## 3 Results

### 3.1 Search results and study selection

**Fig. 1:**
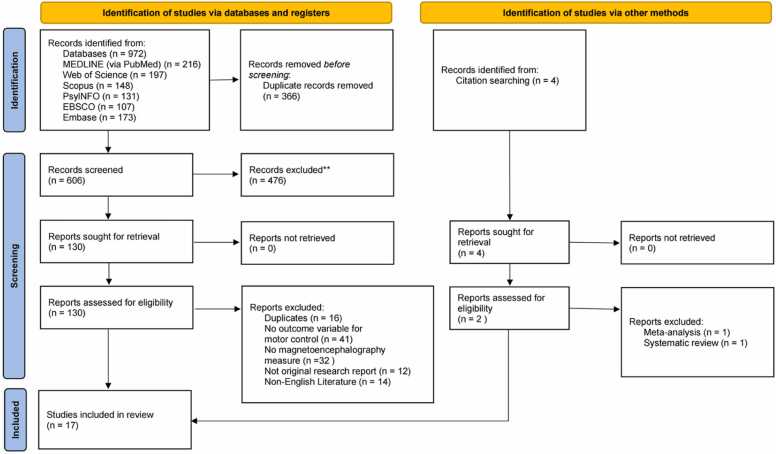
PRISMA flowchart diagram. From Page et al. (2021). For more information, visit http://www.prisma-statement.org/ (Accessed on the 15 April 2024).

A total of 976 articles were identified through the systematic literature search across multiple databases (MEDLINE: 216; Web of Science: 197; Scopus: 148; PsycINFO: 131; EBSCO: 107; Embase: 173; other sources: 4). After removing duplicates, 606 unique articles remained and were screened based on their titles and abstracts. Of these, 476 articles were excluded for not meeting the inclusion criteria. The remaining 130 articles underwent full-text assessment, leading to the exclusion of 115 records for the following reasons: duplicate records (n = 16), no motor control outcome variables (n = 41), absence of MEG measures (n = 32), not original research (n = 12), and non-English language publications (n = 14). Two additional studies were included after excluding meta-analyses (n = 1) and systematic reviews (n = 1) through citation searching. In total, 17 articles met the inclusion criteria and were included in this systematic review. The complete literature search process is illustrated in Fig. 1.

### 3.2 Study characteristics

An overview of the study characteristics is provided in Table 3. All studies employed a cross-sectional design. A total of 744 participants were included across the 17 studies, with 303 women (40.73 %), although two studies did not report gender information (Kurz et al., 2016, Kamp et al., 2013). One study was excluded from the review as it did not align with the research theme (Walshe et al., 2023). The age of participants ranged from children to older adults. Children and adolescents were aged 4–19 years, with an average range of 11–16 years (Gaetz et al., 2010, Kurz et al., 2016, Wilson et al., 2010, Heinrichs-Graham et al., 2020, Fung et al., 2022). Young adults were between 24 and 36 years old, with an average range of 25–28 years (Gehringer et al., 2019b, Burianova et al., 2020, Walker et al., 2020). Older adults were 65 years and older (Heinrichs-Graham and Wilson, 2016, Mary et al., 2015). Regarding motor dominance, left-sided dominance was reported in a total of 21 participants across four studies (Heinrichs-Graham et al., 2020, Fung et al., 2022, Trevarrow et al., 2019, Killanin et al., 2023), while four studies did not report this information (Heinrichs-Graham et al., 2018a, Heinrichs-Graham and Wilson, 2016, Kurz et al., 2016, Gaetz et al., 2020). In the remaining studies, participants were predominantly right-side dominant. One study compared individuals with Autism Spectrum Disorder (ASD) to a healthy control group; for this review, only the data from the healthy group were included (Gaetz et al., 2020). This broad range of participants provided a comprehensive representation of different age groups.

**Table 3:** Characteristics of the studies.

**Reference**	**Country of Origin**	**Journal**	**Participants**		**MEG Measurement**				**Study Design**
			(Age, F - female)	Dominant side	Equipment Type	Number of Channels	Sampling Rate (Hz)	Data Analysis Method	
(Gaetz et al., 2010)	United States and Canada (Children's Hospital of Philadelphia, Hospital for Sick Children, Toronto)	NeuroImage	10 young children (4 – 6 yrs, 5 F), 10 adolescent children (11 – 13 yrs, 5 F), 10 adults (24 – 42 yrs, 5 F)	All right-handed	CTF MEG system (VSM MedTech, Vancouver, Canada)	151	625	Beamforming; Morlet wavelet transform	CSD
(Wilson et al., 2010)	United States (University of Nebraska Medical Center, University of Colorado Denver School of Medicine)	Brain and Cognition	10 typically-developing children and adolescents (8 – 15 yrs, 6 F)	All right-handed	Magnes 3600 WH (4-D Neuroimaging, San Diego, CA, USA)	248	508	Linearly-constrained minimum variance; Complex demodulation;	CSD
(Trevarrow et al., 2019)	United States (University of Nebraska Medical Center, Omaha, NE)	NeuroImage	94 children and adolescents (9–15 yrs, 46 F)	5 left-handed	Elekta/MEGIN MEG system (Elekta, Helsinki, Finland)	306	1000	Linearly-constrained minimum variance; Complex demodulation;	CSD
(Heinrichs-Graham et al., 2020)	United States (University of Nebraska Medical Center, Omaha, NE, and other collaborating institutions)	Cerebral Cortex	107 children and adolescents (9–15 yrs, 55 F)	8 left-handed	Elekta/MEGIN MEG system (Elekta, Helsinki, Finland)	306	1000	Linearly-constrained minimum variance; Complex demodulation	CSD
(Kurz et al., 2016)	United States (University of Nebraska Medical Center, Omaha, NE)	Brain Topography	20 children and adolescents (11–19 yrs, NR)	Not reported	Elekta/MEGIN MEG system (Elekta, Helsinki, Finland)	306	1000	Beamforming; Complex demodulation;	CSD
(Killanin et al., 2023)	United States (University of Nebraska Medical Center, Omaha, NE) and other institutions in the US	Developmental Cognitive Neuroscience	68 children and adolescents (9–16 yrs, 35 F)	4 left-handed	Elekta/MEGIN system (MEGIN, Helsinki, Finland)	306	1000	Linearly-constrained minimum variance; Complex demodulation	CSD
(Fung et al., 2022	United States (Institute for Human Neuroscience, Boys Town National Research Hospital, Boys Town, NE)	NeuroImage	69 children and adolescents (mean age: 13.05 yrs, SD: ± 1.74 yrs, 37 F)	4 left-handed	Elekta/MEGIN system (MEGIN, Helsinki, Finland)	306	1000	Complex demodulation; linearly-constrained minimum variance	CSD
(Gehringer et al., 2019a)	United States (University of Nebraska Medical Center, Omaha, NE)	Journal of Physiology	21 adolescents (mean age: 14.0 yrs, SD: ± 2.1 yrs, 9 F), 22 adults (mean age: 36.6 yrs, SD: ± 5.0 yrs, 12 F)	All right-handed	Elekta/MEGIN MEG system (Elekta, Helsinki, Finland)	306	1000	Beamforming with minimum variance beamformer; Complex demodulation	CSD
(Gehringer et al., 2019b)	United States (University of Nebraska Medical Center, Omaha, NE)	Scientific Reports	19 adolescents (mean age: 14.8 yrs, SD: ± 2.5 yrs, 9 F), 19 adults (age: 36.8 yrs, SD: ± 5.0 yrs, 9 F)	All right-handed	Elekta/MEGIN MEG system (Elekta, Helsinki, Finland)	306	1000	Beamforming with minimum variance beamformer; Complex demodulation	CSD
(Heinrichs-Graham et al., 2018a)	United States (University of Nebraska Medical Center, Omaha, NE) and other institutions in the US	Developmental Cognitive Neuroscience	18 children and adolescents (mean: 11.33 yrs, SD: ± 1.61 yrs, 0 F), 16 younger adults (mean: 28.31 yrs, SD: ± 5.44 yrs, 0 F).	Not reported	Elekta/MEGIN MEG system (Elekta, Helsinki, Finland)	306	1000	Linearly-constrained minimum variance; Complex demodulation	CSD
(Gaetz et al., 2020)	United States (Children’s Hospital of Philadelphia, Philadelphia, PA) and UK (University of Plymouth, Devon)	NeuroImage	63 children and young adults (mean: 14.7 yrs, SD: ± 4.9 yrs, 8.2–24.7 yrs, 17 F)	Not reported	CTF MEG system (CTF Inc., Coquitlam, BC, Canada)	275	1200	Synthetic aperture magnetometry beamformer; Hilbert transform	CSD
(Heinrichs-Graham and Wilson, 2016)	United States (University of Nebraska Medical Center, Omaha, NE)	NeuroImage	16 younger adults (mean: 28.31 yrs, SD: 5.44 yrs, 0 F), 17 older adults (mean: 65.41 yrs, SD: ± 7.09 yrs, 0 F)	Not reported	Elekta/MEGIN MEG system (Elekta, Helsinki, Finland)	306	1000	Linearly-constrained minimum variance; Complex demodulation	CSD
(Kamp et al., 2013)	Germany (University of Düsseldorf, Düsseldorf)	AGE	27 participants (mean: 47.9 yrs, overall age ranged between 22 and 77 yrs, NR)	All right-handed	Elekta/MEGIN MEG system (Elekta, Helsinki, Finland)	122/306	1000	Dynamic imaging of coherent sources; Fourier transforms	CSD
(Burianova et al., 2020)	United Kingdom (Swansea University) and Australia (University of Queensland, Macquarie University)	Neuropsychologia	16 young adults (mean: 26 yrs, SD: 4.3 yrs, 8 F), 16 older adults (mean: 64 yrs, SD: ± 4.5 yrs, 8 F)	All right-handed	KIT-Macquarie MEG160 system (Kanazawa Institute of Technology, Kanazawa, Japan)	160	1000	Linearly-constrained minimum variance; Morlet wavelet transform	CSD
(Xifra-Porxas et al., 2019)	Canada (McGill University, Montreal, Quebec) and Spain (Universidad Politécnica de Madrid)	NeuroImage	12 young adults (mean: 24.2 yrs, SD: 2.8 yrs, 4 F), 12 older adults (mean: 67.7 yrs, SD: ± 3.7 yrs, 3 F)	All right-handed	CTF MEG system (CTF Inc., Coquitlam, BC, Canada)	275	2400	Beamforming; Morlet wavelet transform	CSD
(Walker et al., 2020)	Finland (University of Jyväskylä, Aalto University)	Frontiers in Aging Neuroscience	11 young adults (mean: 25 yrs, SD: 3 yrs, 8 F), 12 older adults (mean: 70 yrs, SD: ± 3 yrs, 7 F)	All right-handed	Elekta/MEGIN Neuromag TRIUX system (Elekta Neuromag® TRIUX™, Elekta Oy, Helsinki, Finland)	306	1000	Beamforming; Hilbert transform	CSD
(Mary et al., 2015)	Belgium (Université Libre de Bruxelles, Brussels)	NeuroImage	15 young adults (mean: 24.26 yrs, SD: 3.33 yrs, 7 F), 14 older adults (mean: 69.1 yrs, SD: ± 1.46 yrs, 8 F)	All right-handed	Elekta/MEGIN MEG system (Elekta, Helsinki, Finland)	306	1000	Dynamic statistical parametric mapping; Morlet wavelet transformation	CSD

**Note.** CSD, cross-sectional design.

The studies were published in a variety of reputable journals, including *NeuroImage* (n = 7) (Gaetz et al., 2010, Heinrichs-Graham and Wilson, 2016, Fung et al., 2022, Mary et al., 2015, Trevarrow et al., 2019, Gaetz et al., 2020, Xifra-Porxas et al., 2019), *Brain and Cognitio*n (n = 1) (Wilson et al., 2010), *Cerebral Cortex* (n = 1) (Heinrichs-Graham et al., 2020), *Developmental Cognitive Neuroscience* (n = 2) (Heinrichs-Graham et al., 2018a, Killanin et al., 2023), *Scientific Reports* (n = 1) (Gehringer et al., 2019b), *Frontiers in Aging Neuroscience* (n = 1) (Walker et al., 2020), *Journal of Physiology* (n = 1) (Gehringer et al., 2019a), *Brain Topography* (n = 1) (Kurz et al., 2016), *AGE* (n = 1) (Kamp et al., 2013), and *Neuropsychologia* (n = 1) (Burianova et al., 2020).

The studies were conducted in various countries, primarily in the United States (n = 11) (Heinrichs-Graham et al., 2018a, Heinrichs-Graham and Wilson, 2016, Kurz et al., 2016, Wilson et al., 2010, Heinrichs-Graham et al., 2020, Fung et al., 2022, Gehringer et al., 2019b, Trevarrow et al., 2019, Killanin et al., 2023, Gaetz et al., 2020, Gehringer et al., 2019a), with others from Canada (n = 2) (Gaetz et al., 2010, Xifra-Porxas et al., 2019), the United Kingdom (n = 2) (Burianova et al., 2020, Gaetz et al., 2020), Germany (n = 1) (Kamp et al., 2013), Belgium (n = 1) (Mary et al., 2015), Finland (n = 1) (Walker et al., 2020), Spain (n = 1) (Xifra-Porxas et al., 2019), and Australia (n = 1) (Burianova et al., 2020). Some studies were collaborative efforts between institutions in different countries, such as the United States and Canada (Gaetz et al., 2010), and the United Kingdom and Australia (Burianova et al., 2020).

The studies utilized a range of MEG systems, with the most commonly used being the Elekta/MEGIN system (n = 12) (Heinrichs-Graham et al., 2018a, Heinrichs-Graham and Wilson, 2016, Kurz et al., 2016, Kamp et al., 2013, Heinrichs-Graham et al., 2020, Fung et al., 2022, Gehringer et al., 2019b, Walker et al., 2020, Mary et al., 2015, Trevarrow et al., 2019, Killanin et al., 2023, Gehringer et al., 2019a). Other systems included the CTF MEG system (n = 3) (Gaetz et al., 2010, Gaetz et al., 2020, Xifra-Porxas et al., 2019), the Magnes 3600 WH (n = 1) (Wilson et al., 2010), and the KIT-Macquarie MEG system (n = 1) (Burianova et al., 2020). The number of channels used varied from 122 to 306, and the sampling rates ranged from 508 Hz to 2400 Hz. In Kamp et al.'s study, the system was upgraded from 122 channels to 306 channels mid-study (Kamp et al., 2013).

MEG data analysis methods varied based on the analytical objectives. Source localization techniques, such as Beamforming (n = 6) (Gaetz et al., 2010, Kurz et al., 2016, Gehringer et al., 2019b, Walker et al., 2020, Xifra-Porxas et al., 2019, Gehringer et al., 2019a), Linearly-constrained minimum variance (n = 6) (Heinrichs-Graham et al., 2018a, Heinrichs-Graham and Wilson, 2016, Wilson et al., 2010, Heinrichs-Graham et al., 2020, Trevarrow et al., 2019, Killanin et al., 2023), Synthetic aperture magnetometry (n = 1) (Gaetz et al., 2020) and Dynamic statistical parametric mapping (n = 1) (Mary et al., 2015), were employed to estimate the spatial origins of neural activity. Time-frequency analysis methods, including the Complex demodulation (n = 9) (Heinrichs-Graham et al., 2018a, Heinrichs-Graham and Wilson, 2016, Kurz et al., 2016, Wilson et al., 2010, Heinrichs-Graham et al., 2020, Gehringer et al., 2019b, Trevarrow et al., 2019, Killanin et al., 2023, Gehringer et al., 2019a), Morlet wavelet transform (n = 4) (Gaetz et al., 2010, Burianova et al., 2020, Mary et al., 2015, Xifra-Porxas et al., 2019), Fourier transforms (n = 1) (Kamp et al., 2013) and the Hilbert transform (n = 2) (Walker et al., 2020, Gaetz et al., 2020), were used to examine the temporal dynamics and frequency characteristics of brain signals. Statistical mapping and coherence imaging techniques, such as Dynamic statistical parametric mapping (n = 1) (Mary et al., 2015) and Dynamic imaging of coherent sources (n = 1) (Kamp et al., 2013), enabled the visualization and quantification of coherent brain activity.

### 3.3 Quality and risk-of-bias assessment

Based on the AXIS tool assessment for risk of bias (see Table 4), sixteen studies were categorized as having a very low risk of bias (Gaetz et al., 2010, Heinrichs-Graham et al., 2018a, Heinrichs-Graham and Wilson, 2016, Kamp et al., 2013, Wilson et al., 2010, Heinrichs-Graham et al., 2020, Fung et al., 2022, Gehringer et al., 2019b, Burianova et al., 2020, Walker et al., 2020, Mary et al., 2015, Trevarrow et al., 2019, Killanin et al., 2023, Gaetz et al., 2020, Xifra-Porxas et al., 2019, Gehringer et al., 2019a). The primary reasons for the minor deductions in their scores included small sample size (n < 30) (Gaetz et al., 2010, Wilson et al., 2010, Walker et al., 2020, Mary et al., 2015, Xifra-Porxas et al., 2019), missing or insufficient participant screening criteria (Gaetz et al., 2010, Kamp et al., 2013, Gehringer et al., 2019b, Gehringer et al., 2019a), and not reporting the subject's motor dominant side (Heinrichs-Graham et al., 2018a, Heinrichs-Graham and Wilson, 2016, Gaetz et al., 2020). One study was rated as low risk (Kurz et al., 2016), largely due to these same factors being present simultaneously. No study was deemed to have a high risk of bias. Overall, the methodological quality of the included studies was solid and relatively homogeneous, suggesting that the findings across these 17 studies are both credible and reliable. This consistency in quality underpins the strength of our conclusions and increases confidence in the overall results of this review.

**Table 4:** Quality assessment of the included studies.

Questions Studies Studies	(Gaetz et al., 2010)	(Wilson et al., 2010)	(Trevarrow et al., 2019)	(Heinrichs-Graham et al., 2020)	(Kurz et al., 2016)	(Killanin et al., 2023)	(Fung et al., 2022)	(Gehringer et al., 2019a)	(Gehringer et al., 2019b)	(Heinrichs-Graham et al., 2018a)	(Gaetz et al., 2020)	(Heinrichs-Graham and Wilson, 2016)	(Kamp et al., 2013)	(Burianova et al., 2020)	(Xifra-Porxas et al., 2019)	(Walker et al., 2020)	(Mary et al., 2015)
Q1	1	1	1	1	1	1	1	1	1	1	1	1	1	1	1	1	1
Q2	1	1	1	1	1	1	1	1	1	1	1	1	1	1	1	1	1
Q3	0	0	1	1	0	1	1	1	1	1	1	1	1	1	0	0	0
Q4	1	1	1	1	1	1	1	1	1	1	1	1	1	1	1	1	1
Q5	1	1	1	1	1	1	1	1	1	1	1	1	1	1	1	1	1
Q6	0	1	1	1	0	1	1	0	0	1	1	1	0	1	1	1	1
Q7	1	1	1	1	1	1	1	1	1	1	1	1	1	1	1	1	1
Q8	1	1	1	1	1	1	1	1	1	1	1	1	1	1	1	1	1
Q9	1	1	1	1	1	1	1	1	1	1	1	1	1	1	1	1	1
Q10	1	1	1	1	1	1	1	1	1	1	1	1	1	1	1	1	1
Q11	1	1	1	1	1	1	1	1	1	1	1	1	1	1	1	1	1
Q12	1	1	1	1	0	1	1	1	1	0	0	0	1	1	1	1	1
Q13	1	1	1	1	1	1	1	1	1	1	1	1	1	1	1	1	1
Q14	1	1	1	1	1	1	1	1	1	1	1	1	1	1	1	1	1
Q15	1	1	1	1	1	1	1	1	1	1	1	1	1	1	1	1	1
Q16	1	1	1	1	1	1	1	1	1	1	1	1	1	1	1	1	1
Q17	1	1	1	1	1	1	1	1	1	1	1	1	1	1	1	1	1
Q18	1	1	1	1	1	1	1	1	1	1	1	1	1	1	1	1	1
Q19	1	1	1	1	1	1	1	1	1	1	1	1	1	1	1	1	1
Q20	1	1	1	1	1	1	1	1	1	1	1	1	1	1	1	1	1
Scores	18	19	20	20	17	20	20	19	19	19	19	19	19	20	19	19	19

**Note.** 1, criterion met; 0, criterion not met.

### 3.4 Summary of findings

Across the 17 studies, a wide variety of movement tasks were employed, including visually-cued finger abduction (n = 2) (Gaetz et al., 2010); Trevarrow et al. (2019)), finger flexion-extension (n = 2) (Wilson et al., 2010, Mary et al., 2015), complex finger-tapping (n = 3) (Heinrichs-Graham et al., 2018a, Heinrichs-Graham and Wilson, 2016, Heinrichs-Graham et al., 2020), isometric tasks (n = 4) (Kurz et al., 2016, Kamp et al., 2013, Gehringer et al., 2019b, Gehringer et al., 2019a), motor sequencing (n = 1) (Killanin et al., 2023), button-press tasks (n = 1) (Gaetz et al., 2020), proprioceptive stretch reflex tasks (n = 1) (Walker et al., 2020), handgrip tasks (n = 1) (Xifra-Porxas et al., 2019), and finger configuration tasks (n = 1) (Burianova et al., 2020).

The behavioral outcome variables measured across the 17 studies included several key metrics: movement duration (n = 7) (Gaetz et al., 2010, Heinrichs-Graham et al., 2018a, Heinrichs-Graham and Wilson, 2016, Heinrichs-Graham et al., 2020, Gehringer et al., 2019b, Mary et al., 2015, Killanin et al., 2023), reaction time (n = 8) (Heinrichs-Graham et al., 2018a, Kurz et al., 2016, Heinrichs-Graham et al., 2020, Fung et al., 2022, Gehringer et al., 2019b, Walker et al., 2020, Killanin et al., 2023, Gehringer et al., 2019a), movement accuracy (n = 8) (Heinrichs-Graham et al., 2020, Fung et al., 2022, Burianova et al., 2020, Mary et al., 2015, Trevarrow et al., 2019, Killanin et al., 2023, Xifra-Porxas et al., 2019, Gehringer et al., 2019a), task performance (n = 4) (Kamp et al., 2013, Gehringer et al., 2019b, Walker et al., 2020, Gehringer et al., 2019a), testosterone levels (n = 2) (Fung et al., 2022, Killanin et al., 2023), and force production (n = 3) (Kamp et al., 2013, Gehringer et al., 2019b, Walker et al., 2020). One study did not report specific behavioral outcomes (Wilson et al., 2010).

**Fig. 2:**
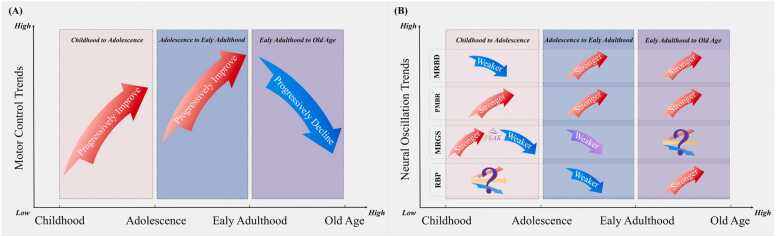
Summary of motor control and neural oscillation developmental trends. (A) Motor control trends over lifespan trajectory, Red arrow = Improve, Blue arrow = Decline; (B) Neural oscillation trends over lifespan trajectory, Red arrow = Stronger, Blue arrow = Weaker, Purple arrow = Weaker (Inconsistency of evidence), Purple question mark = Insufficient research at present, PEAK = MRGS reaches its peak during adolescence, stronger MRBDs = decreases in power relative to baseline, weaker MRBDs = increases in power relative to baseline. This figure outlines lifespan trends in neural oscillations, but further research is needed to clarify specific developmental patterns at each stage.

The neuronal outcome variables across the 16 studies included several key measurements. MRBD (n = 14) (Gaetz et al., 2010, Heinrichs-Graham et al., 2018a, Heinrichs-Graham and Wilson, 2016, Kurz et al., 2016, Wilson et al., 2010, Heinrichs-Graham et al., 2020, Fung et al., 2022, Gehringer et al., 2019b, Burianova et al., 2020, Walker et al., 2020, Trevarrow et al., 2019, Killanin et al., 2023, Gaetz et al., 2020, Xifra-Porxas et al., 2019, Gehringer et al., 2019a), PMBR (n = 9) (Gaetz et al., 2010, Heinrichs-Graham et al., 2018a, Heinrichs-Graham and Wilson, 2016, Wilson et al., 2010, Fung et al., 2022, Walker et al., 2020, Trevarrow et al., 2019, Gaetz et al., 2020, Xifra-Porxas et al., 2019), MRGS (n = 6) (Gaetz et al., 2010, Wilson et al., 2010, Fung et al., 2022, Gehringer et al., 2019b, Trevarrow et al., 2019, Gaetz et al., 2020), resting beta power (n = 5) (Heinrichs-Graham and Wilson, 2016, Burianova et al., 2020, Walker et al., 2020, Mary et al., 2015, Xifra-Porxas et al., 2019), spontaneous beta activity (n = 2) (Heinrichs-Graham et al., 2018a, Burianova et al., 2020), cortico-muscular coherence (n = 1) (Kamp et al., 2013), movement-related theta synchronization (n = 1) (Fung et al., 2022), alpha and beta event-related synchronization (n = 1) (Gehringer et al., 2019b). Overall trends in motor control and neural oscillations with lifespan trajectory are shown in Fig. 2.

#### 3.4.1 Changes from childhood to adolescence

This section includes seventeen studies that investigate behavioral and neuronal changes in motor control from childhood to adolescence (see Table 5). The Behavioral Outcome Variables across these studies include movement duration (Gaetz et al., 2010, Heinrichs-Graham et al., 2020, Killanin et al., 2023), reaction time (Kurz et al., 2016, Heinrichs-Graham et al., 2020, Killanin et al., 2023), testosterone levels (Fung et al., 2022, Killanin et al., 2023), and movement accuracy (Gaetz et al., 2010, Kurz et al., 2016, Fung et al., 2022, Trevarrow et al., 2019). Additional variables, such as time to match target (Kurz et al., 2016) and testosterone levels (Killanin et al., 2023), were also assessed, all reflecting improvements in motor control and efficiency with age. Across the studies, movement duration and reaction time consistently decreased with age, indicating enhanced motor efficiency and quicker processing in older children (Gaetz et al., 2010, Kurz et al., 2016, Heinrichs-Graham et al., 2020, Killanin et al., 2023). Movement accuracy remained stable across all age groups, with participants maintaining a high level of performance (∼98 %) in visually-cued tasks (Trevarrow et al., 2019). Older children showed faster responses in tasks requiring target force matching, reflecting better motor control (Kurz et al., 2016). Additionally, higher testosterone levels in older adolescents were linked to faster reaction times and shorter movement durations, suggesting hormonal influences on motor performance during this developmental stage (Fung et al., 2022, Killanin et al., 2023).

**Table 5:** Motor control from childhood to adolescence.

**Study**	**Movement Task**	**Behavioral Outcome Variables**	**Behavioral Main Findings**	**Neuronal Outcome Variables**	**Neuronal Main Findings**
(Gaetz et al., 2010)	visually-cued finger abduction task	Movement Duration	Younger children (4–6 years) had significantly longer movement durations compared to older children (11–13 years) and adults.	MRBD	The strength and duration of beta ERD were greater in older participants compared to younger participants. ERD was observed in both contralateral and ipsilateral sensorimotor areas in response to motor tasks.
				PMBR	The study observed that PMBR was most pronounced in adults, progressively decreasing in adolescent children (ages 11–13), and was significantly diminished in young children (ages 4–6).
		Accuracy	No significant differences in accuracy were observed across groups.	MRGS	Gamma synchrony, a marker of motor cortical activity, was observed across all age groups, but peak MRGS power was higher in adolescent children compared to adults or younger children.
(Wilson et al., 2010)	index finger flexion-extension movements task	Not reported	Not reported	MRBD	MRBD was observed in the contralateral M1, bilateral SMA, and cerebellum. Significant negative correlations with age were observed during left-hand finger movements (non-dominant hand) in the right SMA (r = −0.58) and left cerebellar lobule IV-V (r = −0.57). These results indicate that the MRBD response became weaker (closer to zero) with increasing age.
				PMBR	PMBR was observed primarily in the contralateral M1 and S1, as well as in the bilateral cerebellum. During left-hand (non-dominant hand) finger movements, PMBR in the right M1 showed a significant negative correlation with age, indicating stronger PMBR responses with increasing age. No significant age-related changes were observed in the SMA or cerebellum.
				MRGS	MRGS was primarily observed in the contralateral M1 and peaked within a 250 ms window following movement onset. Peak MRGS power was highest in the 11- to 13-year-old group compared to younger children. No significant age-related changes were observed in children younger than 11 years.
(Trevarrow et al., 2019)	visually-cued finger abduction task	Accuracy	Accuracy remained consistently high (∼98 %) across all ages.	MRBD	Beta ERD was observed before and during movement, primarily localized in the contralateral sensorimotor cortex. The strength of beta ERD was not correlated with age but showed a significant negative correlation with baseline beta power, indicating stronger ERD in participants with higher spontaneous beta activity.
				PMBR	PMBR was observed primarily in the contralateral M1 and peaked within 0.6–0.9 seconds after movement termination. PMBR occurred after movement termination and was localized in the contralateral sensorimotor cortex. The strength of the PMBR was positively correlated with age, indicating that older participants had stronger PMBR responses.
		Reaction Time	No significant differences in reaction time were observed across groups.	MRGS	A gamma ERS (∼74–84 Hz) was observed during movement execution, localized in the contralateral primary motor cortex. The strength of the gamma ERS was negatively correlated with age, meaning younger participants exhibited stronger gamma responses. MRGS strength was negatively correlated with baseline gamma power, indicating weaker MRGS in those with higher spontaneous gamma activity.
(Heinrichs-Graham et al., 2020)	complex finger-tapping task	Accuracy	Older participants were more accurate.	MRBD	Motor Planning: Beta ERD was observed during the planning phase (−0.4 to 0.0 s), particularly in the bilateral parietal cortices and precentral gyri. Age was positively associated with planning-related beta ERD, indicating stronger desynchronization in older participants. Motor Execution: Beta ERD during execution (0.0–0.4 s) was also observed, with the strength of ERD during planning predicting the strength of ERD during execution in the same brain regions. No significant age-related differences were found in execution-related beta activity.
		Reaction Time	Older participants had faster reaction times.	Developmental Effects	Age was significantly associated with beta ERD in the right parietal cortex during both planning and execution phases. Older participants showed stronger beta ERD during these phases, reflecting improved neural efficiency with age. Beta ERD during motor execution in the right parietal cortex was linked to better motor performance, specifically shorter movement durations.
		Movement Duration	Movement durations decreased with age.	Mediation Effects	Beta ERD in the right parietal cortex during planning predicted subsequent beta ERD during execution, and this execution-related beta ERD was associated with shorter movement durations, suggesting better motor performance.
(Kurz et al., 2016)	isometric knee extension task	Reaction Time	Older children had faster reaction times.	MRBD	MRBD was observed during both motor planning and execution stages in the bilateral parietal cortex, precentral gyrus, and SMA. During motor planning, MRBD in the parietal cortex showed a negative correlation with age. In contrast, during early motor execution, MRBD in the parietal cortex showed a positive correlation with age.
		Time to Match Target	Older children took less time to match target forces.		
		Movement Accuracy	Performance was measured by how quickly participants could match target forces; no specific accuracy data regarding errors.		
(Killanin et al., 2023)	complex motor sequencing task	Reaction Time	Older participants had faster reaction times.	MRBD	MRBD was observed during both the motor planning phase (−0.4 to 0 seconds) and the motor execution phase (0–0.4 seconds), with no significant age-related differences. Testosterone mediated the relationship between age and MRBD during motor execution in the left primary motor cortex, with higher testosterone levels linked to stronger MRBD (i.e., more negative beta values).
		Movement Duration	Movement durations were shorter as age increased.		
		Testosterone Levels	Older participants exhibited higher testosterone levels. Higher testosterone levels were associated with faster reaction times.		
(Fung et al., 2022)	visually-evoked button-press task	Accuracy	No significant differences in accuracy were observed across groups.	MRBD	MRBD was primarily observed in the contralateral primary motor cortex during motor tasks. No direct age-related differences in MRBD strength were reported. Testosterone levels mediated the relationship between age and MRBD, with higher testosterone levels associated with weaker MRBD (i.e., beta values closer to zero). Higher baseline beta power predicted stronger MRBD responses (i.e., more negative beta values).
		Reaction Time	Older participants exhibited faster reaction times.	PMBR	PMBR was primarily observed in the left primary motor cortex. No significant effects of age or testosterone levels on PMBR were found, though a trend suggesting age-related increases in PMBR power was noted across motor tasks.
		Testosterone Levels	Older participants exhibited higher testosterone levels. Higher testosterone levels were associated with faster reaction times, and this relationship remained significant after controlling for age.	MRGS	MRGS was primarily observed in the left primary motor cortex, occurring from approximately 50 ms before to 100 ms after movement onset. Higher baseline gamma power was associated with weaker MRGS responses. No significant indirect effects of age on MRGS were found.
				MRTS	MRTS was primarily observed in the left primary motor cortex. Age predicted baseline theta power, with older participants exhibiting lower baseline theta activity. Stronger baseline theta power was associated with weaker MRTS responses. No significant relationship between testosterone levels and MRTS was found.

**Note.** ERD, event-related desynchronization; ERS, event-related synchronization; MRBD, movement-related beta desynchronization; MRGS, movement-related gamma synchronization; PMBR,post-movement beta rebound, MRTS, movement-related theta synchronization.

The Neuronal Outcome Variables measured across the studies include MRBD (Gaetz et al., 2010, Kurz et al., 2016, Wilson et al., 2010, Heinrichs-Graham et al., 2020, Fung et al., 2022, Trevarrow et al., 2019, Killanin et al., 2023), PMBR (Gaetz et al., 2010, Wilson et al., 2010, Heinrichs-Graham et al., 2020, Fung et al., 2022, Trevarrow et al., 2019), and MRGS (Gaetz et al., 2010, Wilson et al., 2010, Fung et al., 2022, Trevarrow et al., 2019). These variables consistently showed age-related changes in neural oscillatory activity. Overall, older participants often demonstrated weaker MRBD (beta values closer to zero) (Kurz et al., 2016, Wilson et al., 2010), though some research noted stronger MRBD (more negative values) (Gaetz et al., 2010, Heinrichs-Graham et al., 2020) or no significant age effects in specific regions or tasks (Fung et al., 2022, Trevarrow et al., 2019, Killanin et al., 2023). PMBR also increased with age, peaking in older children and adolescents, indicating enhanced sensory feedback and motor inhibition processes following movement termination (Gaetz et al., 2010, Wilson et al., 2010, Trevarrow et al., 2019). In contrast, MRGS was more prominent in younger participants, with gamma power peaking in children and decreasing with age (Gaetz et al., 2010); Wilson et al., 2010; Trevarrow et al., 2019). These findings highlight distinct age-related changes in both beta and gamma neural activity during motor tasks.

#### 3.4.2 Changes from adolescence to early adulthood

This section includes four studies examining motor control from adolescence to early adulthood (see Table 6). The Behavioral Outcome Variables across the studies include the number of targets matched (Gehringer et al., 2019a), time to match targets (Gehringer et al., 2019a), target error (Gehringer et al., 2019a), velocity of force production (Gehringer et al., 2019a), reaction time (Heinrichs-Graham et al., 2018a, Gehringer et al., 2019b, Gaetz et al., 2020, Gehringer et al., 2019a), task accuracy (Heinrichs-Graham et al., 2018a, Gehringer et al., 2019b), motor performance (Gehringer et al., 2019b), and movement duration (Heinrichs-Graham et al., 2018a). These variables capture various dimensions of motor control and performance during this developmental period. Across the studies, adults consistently outperformed adolescents in motor tasks. Adults matched more targets and did so more quickly, demonstrating improved motor control and speed with age (Gehringer et al., 2019a). While no significant differences in target error were found between the groups, adults exhibited higher velocity in force production, indicating stronger motor execution (Gehringer et al., 2019a). Reaction times were significantly faster in adults in multiple studies (Heinrichs-Graham et al., 2018a, Gehringer et al., 2019b, Gehringer et al., 2019a), although one study did not find age-related differences in this measure (Gaetz et al., 2020). In more complex tasks, adults displayed higher accuracy than adolescents (Heinrichs-Graham et al., 2018a), and overall motor performance improved with age, as adults produced greater force and completed tasks more efficiently (Gehringer et al., 2019b). Additionally, movement duration was shorter in adults, suggesting greater motor efficiency (Heinrichs-Graham et al., 2018a).

**Table 6:** Motor Control from Adolescence to Adulthood.

**Study**	**Movement Task**	**Behavioral Outcome Varibales**	**Behavioral Main Findings**	**Neuronal Outcome Variables**	**Neuronal Main Findings**
(Gehringer et al., 2019a)	isometric ankle plantarflexion target-matching task	Number of Targets Matched	Adults matched more targets than adolescents.	Alpha Event-Related Desynchronization	No significant differences in alpha ERD were observed between pre- and post-practice sessions in either age group, though adults exhibited generally stronger alpha ERD than adolescents in the parietal and occipital cortices.
		Time to Match Targets	Adults matched targets faster than adolescents.		
		Target Error	No significant difference between adults and adolescents.	MRBD	Adults showed stronger beta ERD in the sensorimotor cortex during both motor planning and execution compared to adolescents. After practice, beta ERD increased in adults but decreased in adolescents, showing an age-related difference in how motor practice affects neural oscillations.
		Velocity of Force Production	Adults produced force at a higher velocity than adolescents.		
		Reaction Time	Adults had faster reaction times than adolescents.		
(Gehringer et al., 2019b)	isometric ankle plantarflexion force task	Task Accuracy	No significant differences in accuracy were found between adults and adolescents.	Alpha-Beta ERS	Adolescents exhibited greater attenuation of alpha-beta ERS during the motor task compared to adults.
		Reaction Time	Adults had significantly faster reaction times.	MRBD	Adults showed greater attenuation of beta ERD during the motor task compared to adolescents.
		Motor Performance	Adults produced more force and matched target forces faster than adolescents.	MRGS	No significant difference in the attenuation of gamma ERS between adolescents and adults during the task.
(Heinrichs-Graham et al., 2018a)	complex finger-tapping task	Accuracy	Youth were significantly less accurate than young adults.	Spontaneous Beta Activity	Younger adults had lower spontaneous beta activity during rest compared to youth.
		Reaction Time	Youth had slower reaction times compared to young adults.	MRBD	Younger adults exhibited stronger beta ERD compared to youth, indicating increased beta suppression during movement.
		Movement Duration	Youth had longer movement durations than young adults	PMBR	Younger adults showed a stronger PMBR compared to youth, reflecting greater resynchronization after movement.
(Gaetz et al., 2020)	visually-evoked button-press task	Reaction Time	There were no significant age-related differences in reaction times.	MRBD	Beta-ERD increased with age, reflecting developmental changes in motor cortical activity.
				PMBR	PMBR increased with age, indicating stronger post-movement beta oscillations in older participants.
				MRGS	MRGS decreased with age, showing a reduction in gamma synchronization as participants got older.

**Note.** ERD, event-related desynchronization; ERS, event-related synchronization; MRBD, movement-related beta desynchronization; MRGS, movement-related gamma synchronization; PMBR,post-movement beta rebound.

The Neuronal Outcome Variables examined include alpha event-related desynchronization (ERD) (Gehringer et al., 2019a), MRBD (Heinrichs-Graham et al., 2018a, Gehringer et al., 2019b, Gaetz et al., 2020, Gehringer et al., 2019a), PMBR (Heinrichs-Graham et al., 2018a, Gaetz et al., 2020), MRGS (Gehringer et al., 2019b, Gaetz et al., 2020), alpha-beta event-related synchronization (ERS) (Gehringer et al., 2019b), and spontaneous beta activity (Heinrichs-Graham et al., 2018a). These variables reflect changes in brain oscillations during motor tasks, revealing age-related differences in neural processing. Alpha ERD was generally stronger in adults than in adolescents, suggesting greater neural engagement in motor tasks (Gehringer et al., 2019a). MRBD was consistently stronger in adults across all phases of motor tasks, with beta desynchronization increasing with age, showing significant differences between adults and adolescents, particularly after motor practice (Heinrichs-Graham et al., 2018a, Gehringer et al., 2019b, Gaetz et al., 2020, Gehringer et al., 2019a). PMBR also increased with age, suggesting enhanced post-movement motor inhibition and sensory feedback processes in adults (Heinrichs-Graham et al., 2018a, Gaetz et al., 2020). MRGS showed mixed results, with some studies finding no significant differences between adults and adolescents, while others observed a decrease in gamma activity with age (Gehringer et al., 2019b, Gaetz et al., 2020). Adolescents exhibited greater attenuation of alpha-beta ERS compared to adults, reflecting differences in oscillatory modulation with age (Gehringer et al., 2019b). Lastly, spontaneous beta activity was lower in adults than in adolescents, suggesting more efficient neural processing in adulthood (Heinrichs-Graham et al., 2018a). These findings highlight significant age-related changes in both beta and gamma neural activity, indicating the maturation of motor control networks from adolescence to early adulthood.

#### 3.4.3 Changes from early adulthood to old age

This section includes seven studies examining motor control from early adulthood to old Age (see Table 7). The behavioral outcome variables across the studies include accuracy (Heinrichs-Graham et al., 2018a, Heinrichs-Graham and Wilson, 2016, Burianova et al., 2020, Xifra-Porxas et al., 2019), reaction time (Heinrichs-Graham et al., 2018a, Heinrichs-Graham and Wilson, 2016), movement duration (Heinrichs-Graham et al., 2018a, Heinrichs-Graham and Wilson, 2016), task performance and fine motor control (Xifra-Porxas et al., 2019), handgrip strength (Xifra-Porxas et al., 2019), dynamic balance performance (Walker et al., 2020), execution time (Mary et al., 2015), EMG power amplitude and frequency (Kamp et al., 2013), and consistency across conditions (Burianova et al., 2020). These variables assess various aspects of motor performance and control, highlighting differences between younger and older adults in different tasks.

**Table 7:** Motor Control from Adulthood to Older Adulthood.

**Study**	**Movement Task**	**Behavioral Outcome Varibales**	**Behavioral Main Findings**	**Neuronal Outcome Variables**	**Neuronal Main Findings**
(Heinrichs-Graham and Wilson, 2016)	complex finger-tapping task	Accuracy	Both groups had over 96 % accuracy with no significant difference between younger and older participants.	MRBD	Older participants showed stronger beta ERD during movement compared to younger participants.
		Reaction Time	No significant difference in reaction times between the age groups.	Resting Beta Power	Higher resting beta power was observed in older participants in both motor regions.
		Movement Duration	Older participants took significantly longer (878.97 ms) to complete the task compared to younger participants (593.00 ms, p < 0.001).	Correlations	Stronger resting beta power was linked to stronger beta ERD, and stronger beta ERD was associated with longer movement duration.
(Heinrichs-Graham et al., 2018a)	complex finger-tapping task	Accuracy	There was no significant difference in accuracy between younger adults and older adults.	Spontaneous Beta Power	Older adults had significantly higher spontaneous beta power in both the left and right precentral gyri compared to younger adults.
		Reaction Time	Younger adults and older adults showed no significant difference in reaction time.	MRBD	Older adults exhibited significantly stronger beta-ERD (greater beta suppression) during movement in both hemispheres compared to younger adults.
		Movement Duration	Younger adults completed the motor sequences significantly faster than older adults.	PMBR	Older adults showed significantly stronger PMBR compared to younger adults, indicating greater resynchronization of beta activity after movement.
(Kamp et al., 2013)	weak to moderate isometric contraction task	EMG power amplitude	There were no significant differences between the age groups.	Cortico-Muscular Coherence	CMC frequency at beta frequency decreased with age, with younger participants exhibiting higher CMC frequencies compared to middle-aged and elderly participants. CMC amplitude increased with age, with elderly participants showing significantly higher CMC amplitudes compared to younger and middle-aged participants. This suggests greater motor cortical involvement in older adults during isometric contraction.
		EMG power frequency	EMG power frequency decreased with age, showing significantly lower frequencies in the middle-aged and elderly groups compared to the younger participants.	M1 Power	M1 power amplitude at beta frequencies increased with age, while M1 power frequency decreased, showing a shift toward lower frequencies in older participants.
				MEG-EMG Phase Lags	There were no significant differences in MEG-EMG phase lags across age groups, indicating that nerve conduction time remained consistent regardless of age.
(Burianova et al., 2020)	Finger Configuration Task	Accuracy	There were no significant differences in task accuracy between younger adults and older adults.	MRBD	Older adults exhibited significantly stronger MRBD compared to younger adults during both motor execution (ME) and motor imagery (MI) tasks, indicating greater beta power reduction in older adults.
		Consistency Across Conditions	There were no significant differences between younger adults and older adults.	Beta Band Activity	Older adults had more pronounced beta desynchronization, which may be linked to age-related changes in GABAergic inhibition.
				Task Performance and Neural Activity	In older adults, stronger MRBD of the sensorimotor network were linked to poorer performance on motor tasks.
(Xifra-Porxas et al., 2019)	Unimanual isometric handgrips; Bimanual steady handgrips	Accuracy	There were no significant differences between younger and older adults during both unimanual and bimanual handgrip tasks.	Resting Beta Power	Older adults exhibited significantly higher resting beta power compared to younger adults, particularly in motor, frontal, and parietal regions.
		Fine Motor Control	Older adults had significantly worse performance on the Nine Hole Peg Test and Purdue Pegboard Test, reflecting declines in fine motor dexterity compared to younger adults.	MRBD	During dynamic hand contractions, older adults showed significantly greater MRBD in frontal, premotor, and motor regions compared to younger adults. No significant age-related differences in MRBD were observed during sustained hand contractions.
				Beta Modulation	Older adults exhibited a more pronounced modulation of beta oscillations during dynamic contractions compared to younger adults, with greater beta suppression observed during movement initiation and dynamic force production.
		Handgrip Strength	No significant differences in handgrip strength between younger and older adults.	PMBR	No significant age-related differences were found in PMBR during either unimanual or bimanual tasks.
(Walker et al., 2020)	Proprioceptive stretch reflex task	Dynamic Balance Performance	Older adults showed significantly poorer balance control, with greater velocity of center-of-pressure (CoP) displacement compared to younger adults. There was no significant age-related difference in total CoP displacement.	MRBD	Older adults exhibited significantly stronger beta suppression in response to proprioceptive stimulation compared to younger adults.
				PMBR	There was no significant difference between older and younger adults.
		Correlation with Beta Suppression	In older adults, poorer balance performance was correlated with stronger beta suppression following proprioceptive stimulation.	Resting Beta Power	Older adults had significantly higher resting beta power compared to younger adults, particularly in motor areas.
				Correlation with Balance Performance	Stronger beta suppression was correlated with poorer balance performance in older adults.
(Mary et al., 2015)	Simple Movement Task (SMT)	Execution Time	There was no significant difference in execution times between younger and older adults during the SMT before and after the learning phase.	Mu-Alpha and Mu-Beta Power	In younger adults, mu-alpha and mu-beta rhythms showed a significantly stronger post-movement rebound after motor learning, indicating enhanced sensorimotor plasticity. Older adults showed a reduced modulation of both mu-alpha and mu-beta rhythms after learning, reflecting diminished neural plasticity compared to younger adults.
	Finger Tapping Task (FTT)	Number of Correct Chunks	Older adults performed the sequence more slowly than younger adults, generating fewer chunks of the sequence per trial.	Post-Movement Rebound	Younger adults had a more pronounced post-movement rebound in both the alpha and beta bands, particularly in the right SM1, after motor sequence learning. Older adults exhibited a weaker rebound, indicating age-related reductions in sensorimotor plasticity.
		Execution Time	Both younger and older adults improved their speed in completing the finger-tapping sequence across trials, indicating motor learning.		
		Performance Boost	Younger adults showed a significant boost in performance during the retest 30 minutes after learning, with faster execution times. In contrast, older adults showed no significant improvement in performance at the retest.	Source Localization	Mu-alpha activity was localized in the postcentral gyrus (somatosensory cortex), and mu-beta activity was localized in the precentral gyrus (motor cortex) in both age groups.

**Note.** ERD, event-related desynchronization; ERS, event-related synchronization; MRBD, movement-related beta desynchronization; MRGS, movement-related gamma synchronization; PMBR,post-movement beta rebound; SM1, primary sensorimotor cortex.

Overall, accuracy remained high and showed no significant differences between younger and older adults across various tasks (Heinrichs-Graham et al., 2018a, Heinrichs-Graham and Wilson, 2016, Burianova et al., 2020, Xifra-Porxas et al., 2019). Similarly, reaction times were consistent across age groups, with no significant differences reported (Heinrichs-Graham et al., 2018a, Heinrichs-Graham and Wilson, 2016). However, older adults demonstrated longer movement durations compared to younger adults in tasks such as finger tapping (Heinrichs-Graham et al., 2018a, Heinrichs-Graham and Wilson, 2016). Regarding fine motor control, older adults performed worse in dexterity tests, though handgrip strength was similar across age groups (Xifra-Porxas et al., 2019). For dynamic balance performance, older adults exhibited poorer balance control, although no differences were observed in overall center-of-pressure displacement (Walker et al., 2020). While execution time was comparable across age groups in simple tasks, younger adults showed a speed advantage following motor learning, unlike older adults (Mary et al., 2015). Additionally, although EMG power amplitude remained consistent, older adults displayed lower EMG power frequency than younger participants (Kamp et al., 2013). Lastly, performance consistency across conditions did not differ significantly between younger and older adults (Burianova et al., 2020). These findings suggest that while some aspects of motor performance, such as movement duration and dexterity, decline with age, others, like accuracy and reaction time, remain stable.

The neuronal outcome variables include MRBD (Heinrichs-Graham et al., 2018a, Heinrichs-Graham and Wilson, 2016, Burianova et al., 2020, Xifra-Porxas et al., 2019), PMBR (Heinrichs-Graham et al., 2018a, Walker et al., 2020, Mary et al., 2015, Xifra-Porxas et al., 2019), and resting beta power (Heinrichs-Graham and Wilson, 2016, Walker et al., 2020, Xifra-Porxas et al., 2019). In addition, spontaneous beta power was measured in older adults (Heinrichs-Graham et al., 2018a), and beta band suppression was noted in response to proprioceptive stimulation (Walker et al., 2020). Cortico-muscular coherence (CMC) (Kamp et al., 2013) and mu-alpha and mu-beta power (Mary et al., 2015) also captured age-related changes in motor cortex activity.

Across the studies, older adults consistently exhibited stronger MRBD than younger adults, reflecting greater reductions in beta power during movement tasks (Heinrichs-Graham et al., 2018a, Heinrichs-Graham and Wilson, 2016, Burianova et al., 2020, Xifra-Porxas et al., 2019). PMBR was also stronger in older adults in several studies, indicating more robust motor inhibition and sensory feedback following movement termination, though the effect was task-dependent and not always significant (Heinrichs-Graham et al., 2018a, Walker et al., 2020, Xifra-Porxas et al., 2019). Resting beta power was consistently higher in older adults, particularly in motor regions, suggesting age-related increases in baseline beta activity (Heinrichs-Graham et al., 2018a, Walker et al., 2020, Xifra-Porxas et al., 2019). Similarly, spontaneous beta power was significantly higher in older adults (Heinrichs-Graham et al., 2018a). Beta band suppression in response to proprioceptive stimulation was stronger in older adults, and this increased suppression was linked to poorer balance performance (Walker et al., 2020). In contrast, CMCamplitude increased with age, while CMC frequency decreased, indicating greater motor cortical involvement but slower processing in older adults (Kamp et al., 2013). Finally, mu-alpha and mu-beta power exhibited less modulation in older adults after motor learning, suggesting reduced neural plasticity compared to younger participants (Mary et al., 2015). These findings highlight both compensatory mechanisms and age-related declines in neural function related to motor control.

## 4 Discussion

The primary objective of this study was to synthesize evidence on developmental changes in MC and neural activity across the lifespan using MEG. We reviewed 17 studies that examined key behavioral outcomes—such as movement duration, reaction time, and accuracy—alongside neural measures like MRBD, PMBR, and MRGS. Our findings revealed improvements in motor efficiency from childhood to early adulthood, followed by declines in motor performance in older adults, accompanied by neural dedifferentiation and reduced specificity. In the following sections, we discuss how these findings relate to age-related changes in motor function and neural processing.

### 4.1 Neural oscillations related to motor control

#### 4.1.1 MRBD and motor planning and execution

Before and during movement, beta oscillations decrease significantly from baseline levels, typically beginning approximately 1.0 second before movement initiation and subsiding shortly thereafter. This decrease, known as MRBD, is closely associated with the planning and execution of motor activities (Tzagarakis et al., 2010, Heinrichs-Graham and Wilson, 2015). Studies employing EEG, MEG, and invasive electrocorticography consistently demonstrate reductions in cortical oscillatory activity within the beta range (15–30 Hz) preceding movement onset, with these changes persisting throughout most motor activities (Jurkiewicz et al., 2006, Kilner et al., 2004, Pfurtscheller et al., 2003).

The underlying mechanism of MRBD involves the activation of localized cortical areas, which enhances both tactile perception and motor output (Pineda, 2005). By facilitating a substantial reduction in beta power, MRBD enables a transition from stable or inhibitory motor states to efficient movement planning and execution (Cheyne, 2013, Kilavik et al., 2013). Interestingly, the localized cortical activation observed near movement onset has been increasingly linked to MRGS (Heinrichs-Graham et al., 2018b, van Wijk et al., 2012), rather than being solely interpreted as a peak in β ERD.

Neurobiochemical research has revealed that increased intracortical gamma-aminobutyric acid (GABA)-ergic inhibition leads to heightened resting beta power, while muscular activity amplifies MRBD during dynamic contractions (Muthukumaraswamy et al., 2013). Additionally, MRBD is linked to bilateral activation in the frontoparietal cortex. Muscle contractions evoke more pronounced beta ERD in contralateral brain regions, with a largely homogeneous topology centered in the anterior and posterior central gyri (Kurz et al., 2016, Wilson et al., 2010). This reflects the brain's coordination of motor planning and execution through desynchronization in the beta band, particularly in regions responsible for sensorimotor integration and movement control.

#### 4.1.2 PMBR and motor termination

PMBR reaches its peak amplitude approximately 0.5–1.0 seconds after the cessation of movement and persists for around 1.0 second before returning to baseline levels (Gaetz et al., 2010, Heinrichs-Graham et al., 2017). Reduced PMBR has been associated with impaired force accuracy and decreased hand flexibility (Arpin et al., 2017, Laaksonen et al., 2012), whereas increased PMBR has been associated with improved motor outcomes following stroke (Parkkonen et al., 2017). This suggests that PMBR serves as a potential cortical marker of sensorimotor function, with MRGS also playing a role in facilitating motor recovery after stroke (Wilson et al., 2011).

PMBR is widely interpreted as a neural mechanism reflecting sensory feedback processing and motor inhibition within the sensorimotor cortex (SMC) following movement termination (Heinrichs-Graham et al., 2017, Wilson et al., 2010). This rebound may reflect active inhibition within cortical networks after movement (Solis-Escalante et al., 2012), convey afferent feedback to the SMC (Houdayer et al., 2006), or facilitate the planning of future motor actions through the execution of internal models (Arpin et al., 2017). Spooner and Wilson (2022) noted that PMBR typically exhibits a broader frequency bandwidth and higher peak frequency compared to MRBD, further indicating its distinct role in post-movement sensory integration and motor control adjustments.

Magnetic resonance spectroscopy studies have shown a positive correlation between PMBR power and inhibitory GABA levels in the SMC, reinforcing its role in motor inhibition (Gaetz et al., 2011). Unlike MRBD, which primarily originates in the precentral gyrus, PMBR is centered in the postcentral gyrus, reflecting a more sensory-driven feedback process (Heinrichs-Graham et al., 2017). Moreover, PMBR involves widespread neural activity, engaging the SMC, premotor cortex, supplementary motor area (SMA), and medial prefrontal cortex, indicating broad neural coordination during post-movement processes (Heinrichs-Graham et al., 2017).

#### 4.1.3 MRGS and motor command activation

A transient gamma synchronization, occurring at frequencies between 60 and 90 Hz, emerges immediately following movement onset and lasts for approximately 50–250 ms (Heinrichs-Graham et al., 2018b, Muthukumaraswamy et al., 2013, Dalal et al., 2008). MRGS is thought to correspond to the activation of initial motor commands, specifically the precise execution signals related to the onset of muscle activity (Cheyne and Ferrari, 2013). This interpretation is reinforced by evidence showing that gamma event-related synchronization (ERS) occurs during active motor tasks but is absent during passive joint movements (Muthukumaraswamy, 2010). MRGS is a key component of the movement-related gamma network, which is sensitive to complex motor control and higher cognitive processes during movement.

Notably, MRGS amplitude and peak frequency are influenced by cognitive-motor response interference (Wiesman et al., 2020, Gaetz et al., 2013); MRGS amplitude decreases as the accuracy of contextual responses improves (Wiesman et al., 2021). Recent studies have also linked MRGS to motor learning, observing a gradual decrease in MRGS as individuals learn and master motor tasks (Walshe et al., 2023). This evidence suggests that MRGS may reflect higher levels of motor control or cognitive processing in response to dynamic environmental demands during movement (Ulloa, 2021). Additionally, a positive correlation has been found between MRGS amplitude and inhibitory GABA concentrations in the SMC, indicating that GABA plays a significant role in regulating both motor cortical beta and gamma rhythms (Gaetz et al., 2011).

### 4.2 Changes in neural oscillations from childhood to adolescence

#### 4.2.1 MRBD overall weakens with age

Behaviorally, children and adolescents aged 9–19 exhibit improvements in motor performance as they age, including enhanced accuracy, faster reaction times, and shorter task durations (Kurz et al., 2016, Heinrichs-Graham et al., 2020). As children transition into adolescence, MC abilities improve, with MRBD in the primary motor cortex (M1) showing minimal age-related changes and MRBD in secondary motor areas, such as the SMA and parietal lobe, gradually weakening. However, the relationship between MRBD during movement planning and execution and motor performance remains complex, with some inconsistencies in the findings.

In a study by Gaetz et al. (2010), MRBD was analyzed during finger movements across children (4–6 years), adolescents (11–13 years), and adults (24–42 years). The study found that the strength and duration of MRBD in the bilateral SMC, including both contralateral and ipsilateral M1, were greater in older participants compared to younger participants, particularly in the contralateral hemisphere. Linear regression analysis indicated a significant relationship between age and MRBD in children and adults, but not in adolescents. However, Gaetz et al. did not report the direction of the regression coefficients, leaving it unclear whether MRBD becomes stronger (i.e., more negative beta values) or weaker (i.e. closer to zero) with increasing age.

Conversely, Wilson et al. (2010) reported age-related changes in MRBD in secondary motor areas during left-hand (non-dominant hand) finger movements in children and adolescents (8–15 years). Significant negative correlations with age were observed in the right SMA (r = -0.58) and left cerebellar lobule IV-V (r = -0.57), indicating that MRBD responses became weaker (i.e., closer to zero) with increasing age. These findings suggest that MRBD in secondary motor areas decreases with age in children and adolescents, potentially reflecting the maturation of motor cortical inhibitory processes during this developmental period. Similarly, Kurz et al. (2016) observed beta oscillatory changes in adolescents (11–19 years) during leg flexion-extension tasks. During motor planning (−0.3 to 0 seconds), MRBD in the superior parietal cortex showed a negative correlation with age, with older adolescents displaying weaker MRBD (i.e., beta values closer to zero). However, during the early phase of leg contraction (0.3–0.6 seconds), MRBD in the same region showed a positive correlation with age, with older adolescents exhibiting stronger MRBD (i.e., more negative beta values). The stronger MRBD observed in younger participants during motor planning may reflect their greater reliance on sensory feedback for motor execution. Trevarrow et al. (2019) examined beta oscillations in 94 children and adolescents (9–15 years) during visual and auditory key-press tasks and found no significant age-related differences in MRBD in the primary motor cortex. This discrepancy could be attributed to smaller sample sizes (10–20 participants) in earlier studies (Gaetz et al., 2010, Kurz et al., 2016, Wilson et al., 2010).

Overall, current evidence suggests that the correlation between age and MRBD in M1 during adolescence is minimal, potentially due to M1's early maturation. However, age seems to influence MRBD differently during the planning and execution phases of movement. MRI studies indicate that the precentral gyrus matures by age 9, while regions within the motor network, such as the parietal cortex, continue to develop into puberty (Heinrichs-Graham et al., 2020, Shaw et al., 2008).

Heinrichs-Graham et al. (2020) conducted a study involving 107 children and adolescents (aged 9–15) performing complex finger-keystroke tasks to examine the relationship between age and MRBD oscillations. The study found a positive correlation between age and task accuracy, alongside negative correlations with reaction time and movement duration. During motor planning (approximately −0.4 to 0 seconds before movement), beta ERD in the right parietal cortex increased with age (i.e., more negative beta values), indicating stronger desynchronization in older participants. No significant age-related differences in beta ERD were observed during execution (0–0.4 seconds after movement onset). Mediation analysis revealed that stronger planning-related beta ERD predicted longer movement durations, whereas stronger execution-related beta ERD predicted shorter movement durations.

These findings highlight the complex role of the right parietal cortex in regulating MRBD's influence on motor task performance, with age-related differences affecting movement duration. The results emphasize the enduring involvement of the right parietal cortex in complex motor processing and the critical role of oscillatory activity in motor control. Consistent with previous research, strong correlations in the right parietal cortex during the planning phase, without extending into the execution phase, may impair performance. Conversely, persistent or increasing oscillatory activity during execution is linked to enhanced performance (Heinrichs-Graham and Wilson, 2015).

In addition, Kurz et al. (2014) found that children with cerebral palsy (ages 10–18) exhibited stronger MRBD in the superior parietal lobe and postcentral gyrus, rather than the precentral gyrus, compared to healthy peers. This abnormal MRBD suggests difficulties in selecting appropriate muscles for coordinated knee extensions in children with cerebral palsy, indicating that stronger MRBD may be associated with poorer motor control.

Furthermore, recent studies have explored the relationship between hormonal changes during puberty and MRBD. Killanin et al. (2023) found that higher testosterone levels during development from childhood to adolescence predicted stronger MRBD in the contralateral M1 during motor execution, with no significant age-related changes observed during motor planning or execution phases. Similarly, Fung et al. (2022) reported that testosterone levels mediated the relationship between age and MRBD, but no direct age-related differences in MRBD were found. Additionally, this study found that higher baseline beta power predicted stronger MRBD responses (i.e., more negative beta values), further emphasizing the complex interplay between hormonal changes and neural oscillatory activity during motor processes. These findings highlight the need for further exploration of how hormonal factors, particularly testosterone, influence the developmental trajectory of MRBD during puberty.

#### 4.2.2 PMBR increased overall with age

The transition from childhood to adolescence is marked by significant improvements in MC, accompanied by an increase in PMBR. Research indicates that PMBR in the motor cortex strengthens with age during this developmental period (Heinrichs-Graham and Wilson, 2015). Trevarrow et al. (2019) found that participants aged 9–15 exhibited stronger PMBR in the left M1 after movement, suggesting that the progressive enhancement of PMBR reflects an improved ability to inhibit motor actions. Magnetic resonance spectroscopy studies have linked PMBR to increased activity of inhibitory interneurons, leading to elevated PMBR intensity (Muthukumaraswamy et al., 2013, Gaetz et al., 2011, Hall et al., 2011). This neurophysiological development plays a crucial role in enhancing motor control.

In contrast, Kurz et al. (2016) observed no significant PMBR following leg flexion and extension movements in children and adolescents aged 11–19, a finding consistent with Gaetz et al. (2010), who reported a lack of significant PMBR in a pediatric group aged 4–6 years. Both studies had relatively small sample sizes (10–20 participants) and large age spans, which may have limited the detection of important changes in PMBR (Gaetz et al., 2010, Kurz et al., 2016). Alternatively, these outcomes could be attributed to the underdeveloped oscillatory mechanisms responsible for autonomous inhibition in younger children. Cheyne et al. (2014) documented PMBR in children aged 3.2–4.8 years during video game–induced finger movements, noting an onset delay of 200–300 ms compared to the more rapid transitions observed in adults. This PMBR latency may reflect the ongoing development of motor control capabilities in children. Studies on adult patients with motor control disorders have similarly observed reduced or delayed PMBR peaks, suggesting a potential link between diminished PMBR and impaired motor control (Robson et al., 2016, Hunt et al., 2019, Barratt et al., 2017, Proudfoot et al., 2017).

Furthermore, Fung et al. (2022) found that PMBR was primarily localized in the left primary motor cortex, with no significant effects of age or testosterone levels on its strength. However, a trend of age-related increases in PMBR power was observed across motor tasks, suggesting a progressive enhancement of PMBR during motor development.

#### 4.2.3 MRGS increases and then decreases with age

During the transition from childhood to adolescence, MRGS follows a developmental trajectory characterized by an initial increase in power, peaking in early adolescence, and subsequently declining with further aging. Variability in MRGS frequency is particularly evident among younger children. Gaetz et al. (2010) found that MRGS power increased in adolescents (11–13 years) compared to younger children (4–6 years), suggesting that MRGS strength reaches its peak during early adolescence before diminishing.

Conversely, Wilson et al. (2010) observed a decrease in MRGS power within the contralateral SMA during finger movements in individuals aged 8–15 years. Similarly, Trevarrow et al. (2019) reported a reduction in MRGS power with age in children and adolescents (9–15 years), along with a negative correlation between baseline resting gamma oscillations and MRGS power. However, neither study found any age-related changes in peak MRGS frequency. These findings indicate that while MRGS power may increase from early childhood to adolescence, it begins to decrease during adolescence.

Cheyne et al. (2014) identified MRGS in children as young as 3.2–4.8 years, noting broad variability in frequency ranges. Some children demonstrated event-related synchronization (ERS) in higher (70–80 Hz) or lower (30–40 Hz) gamma frequency bands, or across both spectra. In contrast, Gaetz et al. (2010) reported MRGS exclusively in higher frequency bands across children, adolescents, and adults, suggesting that MRGS frequencies stabilize in higher bands with increasing age. Despite this, no significant differences in peak gamma frequency were observed between age groups.

Additionally, Gaetz et al. (2020) reported a decrease in MRGS with age in healthy individuals aged 8–24.9 years. Kurz et al. (2014) also observed diminished MRGS at movement onset in children with cerebral palsy, suggesting that abnormal MRBD during movement planning may negatively impact MRGS, thereby affecting motor command execution. Similarly, Fung et al. (2022) found that MRGS was primarily observed in the left M1, with higher baseline gamma power associated with weaker MRGS responses. The study reported no direct age-related differences in MRGS strength, although age was positively associated with testosterone levels, which influence baseline gamma power. These findings suggest that hormonal changes during puberty may contribute to MRGS variability across development.

### 4.3 Changes in neural oscillations from adolescence to early adulthood

#### 4.3.1 MRBD continues growth trend from adolescence to early adulthood

As adolescents progress from puberty into early adulthood, MRBD continues to increase developmentally, particularly during index finger movements based on visual cues. Gaetz et al. (2010) observed that MRBD power increases with age, especially in contralateral M1 compared to ipsilateral M1, during left/right index finger movements when comparing adolescents (11–13 years) to adults (24–42 years).

In a study employing a more complex task, Heinrichs-Graham et al. (2018a) asked participants—juveniles aged 9–14 years and adults aged 20–42 years—to perform a motor sequence task using a five-finger button pad. Results indicated that the juvenile group underperformed compared to adults in task accuracy, reaction time, and movement duration. MEG data revealed higher resting beta oscillations in juveniles during rest with eyes closed, whereas adults exhibited increased MRBD during the task.

Recent findings suggest that resting beta power is tightly linked to MRBD amplitude, with studies showing that higher resting beta levels are associated with stronger MRBD responses (Heinrichs-Graham and Wilson, 2016, Wilson et al., 2014). This association indicates that baseline beta power may set a threshold that must be surpassed to achieve efficient motor planning and execution. Elevated resting beta power in juveniles may partially account for their observed weaker MRBD responses and diminished motor performance during complex tasks. Additionally, the modulation of beta activity may relate to the gradual cortical thinning observed in the motor cortex from childhood through early adulthood, indicating increased neuronal efficiency over this period (Shaw et al., 2008, Vandekar et al., 2015).

In addition to hand-motor evoked responses, Gehringer et al. (2019a) identified age-dependent characteristics of MRBD in adolescents (14.8 ± 2.5 years) and adults (36.6 ± 5 years) during an ankle plantarflexion matching task. Despite both groups showing significant motor performance improvements post-task, adults consistently surpassed adolescents. MEG revealed post-practice MRBD reductions in the SMC during motor planning and execution in adolescents, in contrast to increases observed in adults. This indicates age-related alterations in SMC oscillations following motor task practice, potentially due to task familiarity, variations in GABAergic activity, or developmental differences in the integrity of white matter fiber networks linking relevant cortical areas.

Furthermore, in examining somatosensory cortex processing differences during movement between adolescents (14.8 ± 2.5 years) and adults (36.8 ± 5.0 years), the team investigated motor nerve oscillation changes in participants subjected to electrical tibial nerve stimulation under two conditions: isometric plantarflexion of the ankle (active) and sitting without movement (passive). Results highlighted the emergence of alpha-beta (8–30 Hz, 0–125 ms) ERS and beta (18–26 Hz) oscillatory responses post-stimulation (300–400 ms) across both conditions, with these frequency-specific cortical oscillations being notably weaker during active versus passive states.

Crucially, attenuation of alpha-beta ERS was more pronounced in adolescents, while attenuation of beta ERD was more significant in adults, suggesting differential processing of somatosensory feedback and cortical oscillation modulation across age groups. The pronounced attenuation of alpha-beta ERS in adolescents, as identified by Gehringer et al. (2019b), may stem from movement-related gating mechanisms that hinder the transmission of sensory information to higher cortical areas. This phenomenon indicates that adolescents might face challenges in processing somatosensory feedback during voluntary movements. Conversely, the occurrence of somatosensory beta ERD later in the post-stimulus period could result from a rebound or resetting of cortical oscillations, as suggested by Della Penna et al. (2004) and Palva et al. (2005). Therefore, adolescents might reset somatosensory cortical oscillations during isometric plantarflexion, while adults continue to process somatosensory inputs.

The team further theorized that the observed oscillatory activities might derive from the interaction between sensory information generated through electrical stimulation of Ia afferent nerves and muscle spindles or peripheral alpha motor neurons. This theory aligns with the concept that the H-reflex, modifiable through submaximal electrical stimulation of Ia afferent nerves, evolves in strength throughout adolescence. Such changes in the H-reflex, according to Gehringer et al. (2019b), could correlate with the developmental trajectory of MRBD. Therefore, the modulation of motor performance characteristics during adolescence might partially rely on the alteration of frequency-specific somatosensory cortical oscillations.

Recent investigations have embraced more sophisticated paradigms for exploring changes in human motor-related neural oscillations. Walshe et al. (2023) introduced a simulated driving task requiring participants to navigate acceleration, maintain consistent speed, and brake along a straight roadway equipped with traffic signals. This study revealed an increase in MRBD among individuals aged 16–23, mirroring patterns observed during simpler motor tasks. Notably, MRBD was found to escalate with age, suggesting a uniform progression of MRBD alterations across both simple and complex motor tasks as individuals transition from adolescence into early adulthood.

Furthermore, the study highlighted significant activation within the midline frontal lobe when participants manipulated the accelerator and brake, pointing to enhanced oscillatory activity. This finding potentially indicates the involvement of cognitive control mechanisms in initiating and executing actions. Walshe et al. (2023) propose that future research should extend into examining oscillation characteristics related to the performance of complex motor tasks, thereby broadening our understanding of motor control and its neural underpinnings.

#### 4.3.2 PMBR increases with age

PMBR exhibits a developmental trajectory similar to that of MRBD, with both increasing from adolescence into early adulthood (Gaetz et al., 2010, Heinrichs-Graham et al., 2018a). This age-related progression in PMBR is further corroborated in studies of individuals with autism spectrum disorder (ASD) aged 8–24.9 years, who demonstrate significantly lower PMBR power than their healthy counterparts, with disparities becoming pronounced around 13.2 years of age (Gaetz et al., 2020). The correlation between PMBR and the inhibitory neurotransmitter GABA suggests a developmental delay in ASD, marked by reduced GABA levels in sensorimotor areas (Gaetz et al., 2013, Puts et al., 2017).

Paired-pulse transcranial magnetic stimulation is a method for assessing intracortical inhibition through short-interval cortical inhibition (SICI). It involves applying a pair of stimuli to M1 with a brief interstimulus interval, eliciting motor-evoked potentials (MEPs) in muscles such as the first dorsal interosseous (Garvey and Gilbert, 2004). Studies have shown that SICI more effectively induces MEPs in adults (18–30 years old) compared to juveniles (10–18 years old), suggesting a more mature GABAergic system in adults (Walther et al., 2009, Reis et al., 2002). Further evidence indicates that PMBR is linked to active inhibition within cortical motor networks. Larger PMBR oscillations correspond to greater force outputs during isometric muscle contractions, with rapid movement terminations enhancing PMBR amplitude across sensorimotor and executive control networks (Heinrichs-Graham et al., 2017, Fry et al., 2016). However, existing research predominantly focuses on adult populations (19–30 years old), highlighting the need for more studies on PMBR changes from adolescence to adulthood.

#### 4.3.3 MRGS declines with age

Research into the maturation of motor control abilities reveals an associated decline in MRGS from adolescence to early adulthood, though findings across studies show variability. Gaetz et al. (2010) observed elevated MRGS levels in adolescents (11–13 years) compared to adults (24–42 years), a result potentially influenced by small sample sizes (10 participants) and individual differences. Further analysis by Gaetz et al. (2020) indicated a decrease in MRGS power with age among both healthy individuals and ASD patients aged 8–24.9 years, aligning with earlier adolescent research (Trevarrow et al., 2019). Yet, inconsistencies persist within the current body of research.

Gehringer et al. (2019b) noted that electrical stimulation of the tibial nerve weakened somatosensory cortex gamma event-related synchronization (ERS) during isometric plantarflexion in both adolescent (14.8 ± 2.5 years) and adult (36.8 ± 5.0 years) groups, without significant differences in attenuation levels. This decrease in MRGS could reflect a more focused allocation of attentional resources toward movement control, suggesting MRGS maturation in adolescents.

In a complementary study, Walshe et al. (2023) explored MRGS in participants (16–23 years old) during a complex simulated driving task, finding no clear correlation with age. Participants exhibited a "learning effect," highlighted by significant improvements in task performance over time, paralleled by a gradual reduction in MRGS oscillations. This observation supports the concept that MRGS signifies advanced motor control or cognitive processing capabilities essential for adapting to dynamic environments (Ulloa, 2021).

### 4.4 Changes in neural oscillations from early adulthood to old age

#### 4.4.1 Resting beta power and MRBD increase with aging

The compensation and dedifferentiation hypotheses offer two perspectives on the increased brain recruitment observed in aging. The compensation hypothesis suggests that brain restructuring in older adults compensates for functional declines, while the dedifferentiation hypothesis posits that inefficient recruitment of multiple brain regions results from ambiguous interactions between brain structure and function as individuals age (Reuter-Lorenz and Park, 2010). Current evidence supports the observation that older adults exhibit more pronounced overall oscillations and MRBD compared to younger individuals.

A key study by Burianova et al. (2020) supports the dedifferentiation hypothesis by examining the performance of young adults (26 ± 4.3 years) and older individuals (64 ± 4.5 years) during actual and imagined finger-matching tasks. MEG data showed significantly enhanced MRBD in older adults during both execution and imagination phases. Additionally, fMRI findings revealed increased activation in sensorimotor areas in older adults, with heightened bilateral M1 connectivity negatively correlating with motor execution performance. These findings challenge the compensation hypothesis, suggesting that excessive MRBD and sensorimotor network activation in older adults may reflect a decline in the quality of neural signals with age, leading to reduced motor capabilities (Burianova et al., 2020).

Heinrichs-Graham and Wilson (2016) found that surpassing a specific movement threshold is essential for optimal motor execution. In their study comparing young adults (28.31 ± 5.44 years) and older adults (65.41 ± 7.09 years) during finger movement sequence tasks, no significant differences were observed in button accuracy between the groups, though young adults demonstrated faster reaction times. MEG analysis revealed higher resting beta power in bilateral M1 for older adults compared to younger individuals. Additionally, the study identified a negative correlation between MRBD and movement duration during rest, alongside a positive correlation between power amplitude and MRBD, suggesting that increased MRBD compensates for elevated power levels to facilitate movement.

Heinrichs-Graham and Wilson (2016) demonstrated that effective motor execution requires the motor cortex to achieve a sufficient reduction in beta power (MRBD). In their study comparing young adults (28.31 ± 5.44 years) and older adults (65.41 ± 7.09 years) during finger movement sequence tasks, no significant differences were observed in button accuracy between the groups. However, a significant difference in movement duration was found, with older adults taking longer to complete the sequences, despite similar accuracy levels. MEG analysis revealed higher resting beta power in bilateral M1 for older adults compared to younger individuals. Additionally, the study identified a negative correlation between MRBD and movement duration (indicating that greater MRBD is associated with shorter movement durations), alongside a positive correlation between power amplitude and MRBD, suggesting that increased MRBD compensates for elevated power levels to facilitate movement.

Wilson et al. (2014) noted a diurnal variation in beta power and MRBD, with both intensifying as the day progresses. This pattern aligns with previous studies that found increased GABA concentration enhances resting beta power and MRBD (Muthukumaraswamy et al., 2013). Heinrichs-Graham and Wilson (2016) proposed that the increase in MRBD is driven by elevated resting beta power rather than directly by higher GABA levels, suggesting a complex interaction between neurochemical concentrations and motor function dynamics.

Xifra-Porxas et al. (2019) investigated neural oscillations in young adults (19–28 years) and older adults (60–74 years) during dynamic and sustained grip tasks. The study revealed a notable increase in resting beta power in older adults compared to younger participants. During dynamic contractions, older adults exhibited significantly enhanced MRBD, though no significant differences were observed during sustained contractions. This increase in resting beta power parallels patterns seen in neurodegenerative conditions, such as Parkinson's disease, suggesting that the elevation in beta power may contribute to the decline in fine motor control and cognitive function (Engel and Fries, 2010). Contrary to the compensation hypothesis, which predicts broader MRBD recruitment in older adults, this study found that while MRBD was more generalized in older adults, younger adults demonstrated more focused activation within M1.

A study examining participants aged 9–75 years identified a quadratic relationship between age and resting beta power, with a decrease from adolescence to early adulthood, followed by an increase into older age (Heinrichs-Graham et al., 2018a). Similarly, Rempe et al. (2023) observed a rise in beta power across a wide age range (6–84 years), particularly in the bilateral precentral gyrus. This region, critical for action control, plays a key role in oscillatory activity associated with motor functions (Davis et al., 2012). The age-related increase in beta power during rest may be linked to the evolution of cortical gray matter, which thins rapidly from childhood through adolescence, stabilizes in early adulthood, and thickens slightly in older age, reflecting changes in neural efficiency throughout life (Pfefferbaum et al., 2013). However, Rempe et al. (2022) noted that the increase in sensorimotor cortex beta power in participants aged 22–72 did not correlate with cortical thickness, suggesting that factors beyond cortical structure contribute to these oscillatory changes with age. Interestingly, MRBD exhibits a linear increase from young adulthood to older age, possibly influenced by rising GABA levels over time (Rossiter et al., 2014).

Additionally, Walker et al. (2020) found that older adults experience heightened pain sensitivity before passive ankle rotation compared to younger individuals, along with increased MRBD in response to proprioceptive stimulation. This rise in MRBD was associated with reduced dynamic balance performance, indicating a decline in sensorimotor function with age.

#### 4.4.2 PMBR decreases with aging

Research on PMBR, a key neurophysiological marker linked to motor control, indicates that PMBR tends to decline with advancing age, although findings vary across studies. Liu et al. (2017) observed that older adults (60–78 years) exhibited a significant reduction in PMBR during finger-tapping tasks compared to younger adults (22–35 years). Similarly, Bardouille and Bailey (2019) in a large-scale study involving 567 participants aged 18–88, reported diminished PMBR in older adults, particularly in the hemisphere contralateral to the motor hand, contrasting with patterns seen in younger individuals. This reduction in PMBR is not limited to healthy aging but is also observed in patients with movement disorders, where PMBR decreases after active or passive limb movements, suggesting a link between reduced PMBR and declining motor control abilities in older adults (Arpin et al., 2017, Proudfoot et al., 2017).

PMBR's role extends beyond motor control. It is thought to contribute to active inhibition or reduced excitability within the motor network, possibly through modulation of the GABAergic system, which helps prevent unnecessary movements and improve motor control efficiency (Muthukumaraswamy et al., 2013, Solis-Escalante et al., 2012, Gaetz et al., 2011).

However, not all studies consistently report such declines. Xifra-Porxas et al. (2019) found no significant difference in contralateral hemisphere PMBR between older (60–74 years) and younger adults (19–28 years) during single-handed tasks. Interestingly, an increase in PMBR was noted in the ipsilateral hemisphere, but no significant PMBR differences were observed during two-handed tasks, highlighting the nuanced role of hemisphere engagement in PMBR responses. Similarly, Walker et al. (2020) examined nerve oscillations during passive ankle rotation and found no significant PMBR differences between young (25 ± 3 years) and older participants (70 ± 3 years) post-movement. These inconsistencies suggest a complex interaction between age, movement type (e.g., unilateral/bilateral, active/passive), and PMBR, underscoring the need for further research to clarify the mechanisms by which aging affects PMBR.

Adding to this body of research, Mary et al. (2015) investigated neural oscillations during motor sequence learning in young (18–30 years) and older adults (65–75 years). After two sessions of a simple button-pressing task, older adults demonstrated longer learning times (3000–3500 seconds) in the right primary SM1and showed weaker frequency band rebound in the mu-alpha range, with no significant differences in the mu-beta band rebound. These findings suggest that young adults experience more robust cortical reorganization following motor learning, pointing to a decline in the dynamic plasticity of SM1 with age.

## 5 Limitations and future directions

Research on age-related changes in neural oscillations, particularly concerning gender differences, remains limited. Previous studies have noted gender disparities in neural oscillations, with findings suggesting that men exhibit higher delta, theta, and alpha power compared to women (Rempe et al., 2023, Scheinost et al., 2015, Shaw et al., 2016). Additionally, the influence of circadian rhythms on the development of motor control through neural oscillations is underexplored. Wilson et al. (2014) reported that beta power and MRBD increase across the sensorimotor network as the day progresses, underscoring the potential impact of circadian rhythms on neural dynamics. Future research should investigate how both gender and circadian factors contribute to the development of neural oscillations related to motor control.

Another limitation of the current body of research is the focus on simple motor tasks, which may lack ecological validity. Studies incorporating more complex motor paradigms that simulate real-life motor challenges could provide a more comprehensive understanding of motor control across different age groups. The small sample sizes in several included studies (n < 30) also raise concerns, as they may contribute to inconsistencies in the reported findings (Gaetz et al., 2010, Kurz et al., 2016, Wilson et al., 2010, Walker et al., 2020, Mary et al., 2015, Xifra-Porxas et al., 2019). Furthermore, the dominance of cross-sectional designs restricts the ability to establish causal relationships and track developmental trajectories. To address this, future research should incorporate a wider range of study designs, such as longitudinal studies, which are crucial for improving the credibility and depth of findings. However, a review of the existing literature reveals a notable scarcity of longitudinal studies, likely due to the limited number of researchers in this field, the high costs associated with MEG studies, and the relatively higher costs of conducting longitudinal research.

Moreover, intervention studies addressing age-related declines in motor control are scarce. Emerging research indicates that resting gamma oscillation power follows an age-related trajectory, peaking around 50 years of age before declining (Rempe et al., 2023). Investigating the transitions from early adulthood to midlife and from midlife to older age is essential for developing strategies to prevent neurodegenerative diseases and improve motor function in aging populations. Finally, exploring neuromodulation techniques—such as transcranial electrical or magnetic stimulation and neural feedback training, either independently or in combination—offers a promising avenue for intervention. These approaches could provide novel therapeutic options for addressing motor control disorders associated with development and aging.

## 6 Conclusion

This systematic review synthesizes age-related changes in MC and associated neural oscillations across the lifespan, as measured by MEG. The findings indicate progressive improvements in motor performance from childhood to early adulthood, marked by faster reaction times, shorter movement durations, and increased accuracy, alongside changes in neural oscillatory markers such as weakened MRBD, and increased PMBR and MRGS. While MRBD and PMBR increase with age—improving motor planning, execution, and termination—MRGS peaks in adolescence and decreases thereafter, reflecting shifts in neural mechanisms involved in motor command. In older adults, motor performance declines are accompanied with increased resting beta power and MRBD, which reflect dedifferentiation rather than compensation. These changes suggest inefficient and generalized recruitment of motor-related neural networks, leading to reduced motor specificity and functional declines. However, PMBR diminishes, indicating weakened inhibitory control and sensory feedback processes after movement termination. These changes highlight the complex role of neural oscillations in motor aging and the maintenance of motor function. Future research should explore gender differences, circadian rhythms, middle-aged populations, and more complex motor tasks. Neuromodulation techniques may offer interventions for mitigating age-related declines in motor control, contributing to neurorehabilitation and the prevention of motor decline in aging populations.

## Author contributions

X.Z. (Xinbi Zhang) and M.H. (Mingming Huang) independently screened titles and abstracts from Covidence to determine eligibility for full-text review and subsequently reviewed the full texts of the selected studies. Disagreements were resolved by consensus and in consultation with the third independent author, Y.W. (Yingying Wang). Conceptualization: X.Z. (Xinbi Zhang), M.H. (Mingming Huang), K.W. (Kanya Wongwitwichote), and C.J. (Changhao Jiang); writing—original draft preparation: X.Z. (Xinbi Zhang), M.H. (Mingming Huang), X.Y. (Xiaoxia Yuan), and Y.W. (Yingying Wang); writing—review and editing: X.Z. (Xinbi Zhang), Y.W. (Yingying Wang), K.W. (Kanya Wongwitwichote), and X.Z. (Xiaoke Zhong); supervision: C.J. (Changhao Jiang), S.D. (Shengyu Dai), Y.D. (Yuanfu Dai), and X.Z. (Xiaoke Zhong). All authors, including X.Y. (Xiaoxia Yuan), K.W. (Kanya Wongwitwichote), and S.D. (Shengyu Dai), have read and agreed to the published version of the manuscript.

## Funding

This study receives the support from the 10.13039/501100001809National Natural Science Foundation of China [grant number: 32371132], the Beijing Municipal Social Science Foundation [grant number: 19YTA001], and the Emerging Interdisciplinary Platform for Medicine and Engineering in Sports of the Capital University of Physical Education and Sports.

## CRediT authorship contribution statement

**Xinbi Zhang:** Writing—original draft. **Mingming Huang:** Writing—original draft. **Xiaoxia Yuan:** Writing—original draft. **Xiaoke Zhong:** Supervision. **Shengyu Dai:** Supervision. **Yingying Wang:** Writing—review and editing. **Kanya Wongwitwichote:** Supervision. **Changhao Jiang:** Supervision.

## Declaration of Competing Interest

The authors affirm that there are no existing competing financial interests or personal relationships that could seem to have influenced the findings presented in this manuscript.

## Data Availability

No data was used for the research described in the article.
